# 308. Secondary Infections in Patients Requiring Extracorporeal Membrane Oxygenation (ECMO) for Severe Acute Respiratory Distress Syndrome (ARDS) due to COVID-19 Pneumonia (PNA)

**DOI:** 10.1093/ofid/ofab466.510

**Published:** 2021-12-04

**Authors:** Ryan Rivosecchi, J Alex Viehman, Christina K Thorngren, Ryan K Shields, Fernanda P Silveira, Fernanda P Silveira, Eun Jeong Kwak, Peter Volpe, Vidya Jagadeesan, Cornelius J Clancy, Minh Hong Nguyen, Palash Samanta

**Affiliations:** 1 UPMC Presbyterian Hospital, Pittsburgh, PA; 2 University of Pittsburgh, Pittsburgh, Pennsylvania; 3 University of Pittsburgh Medical Center, Pittsburgh, PA; 4 UPMC, Pittsburgh, Pennsylvania

## Abstract

**Background:**

Rescue ECMO has been used worldwide in patients (pts) with ARDS caused by COVID-19. Bacterial super-infections affect 3.5-14.3% of hospitalized pts with COVID-19. Pts requiring ECMO may be at an increased risk of infection due to their severity of illness, gut translocation and ECMO impact on host immunity.

**Methods:**

This was a retrospective review of pts requiring ECMO for COVID-19 from April 2020-2021 at a single center. Strict definitions of infections (including ventilator-associated PNA, VAP) were in accordance with CDC criteria.

**Results:**

43 ECMO pts with 1065 ECMO days were evaluated. Median age was 53 yrs (range: 21-62) and median BMI was 36.2 (range: 19.4-75.8). 70% were men and 65% were white. 37 patients (86%) experienced a total of 40 infectious episodes with a median onset from ECMO cannulation to first infection of 10.5d (range: 4-50). Median SOFA and SAPSII scores at time of infection were 12 (6-20) and 63 (30-90), respectively. PNA was the most common infection (78%, with 19% of cases complicated by bacteremia and 3% by empyema) (Fig. 1). The most common organisms isolated were Enterobacterales (37%), *S. aureus* (25%) and *P. aeruginosa* (16%) (Fig. 2). Only 2% of all organisms were multi-drug resistant. 3 pts had fungal infections (1 candidemia, 2 aspergillus PNA). Duration of ECMO was significantly longer for infected pts (26d, range: 5-92d) vs (11d, range: 3-24d), p=.01. 95% of infected pts had received steroids vs. 67% of uninfected pts, p=0.09. Treatment success at 1 week was 50%, and 24% and 40% of pts had recurrent infections and persistent/recurrent organisms in clinical cultures, respectively. *S. aureus* (54%) and Enterobacterales (26%) were associated with persistent or recurrent clinical cultures, requiring prolonged antimicrobial therapy. Mortality rate at 30 days was 65% and was significantly higher for pts with infection than those without (67% vs 33%, p=.02).

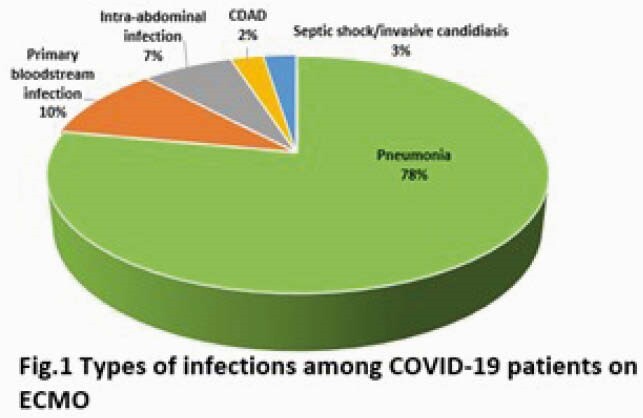

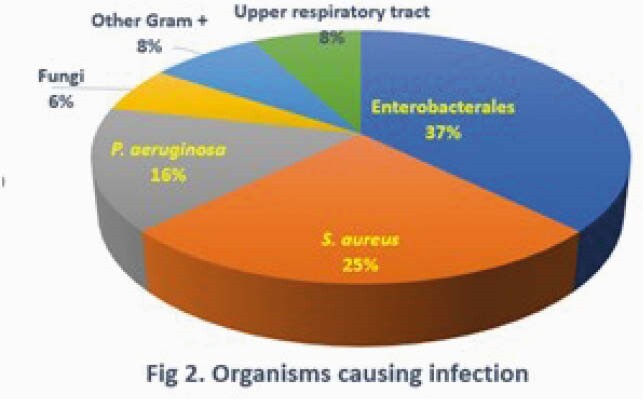

**Conclusion:**

Super-infection (most commonly PNA) occurred in almost all COVID-19 pts requiring ECMO for >4 days, and was a significant risk factor for death. Recurrent infections among survivors were common, especially when caused by Enterbacterales or *S. aureus*. Super-infection and mortality rates of ARDS pts on ECMO for COVID-19 were worse than for ARDS pts on ECMO for influenza at our center.

**Disclosures:**

**Ryan K. Shields, PharmD, MS**, **Shionogi** (Consultant, Research Grant or Support) **Fernanda P. Silveira, MD, MS, FIDSA**, Ansun (Individual(s) Involved: Self): Grant/Research Support; Novartis (Individual(s) Involved: Self): Grant/Research Support; Qiagen (Individual(s) Involved: Self): Grant/Research Support; Shire (Individual(s) Involved: Self): Advisor or Review Panel member, Grant/Research Support; SlieaGen (Individual(s) Involved: Self): Grant/Research Support; Whiscon (Individual(s) Involved: Self): Grant/Research Support **Cornelius J. Clancy, MD**, **Merck** (Grant/Research Support)

